# Cooled Radiofrequency Ablation Versus Cryoneurolysis of the Genicular Nerves for the Symptomatic Pain Management in Knee Osteoarthritis: A Prospective, Randomized, Single-Blinded Clinical Trial

**DOI:** 10.7759/cureus.98323

**Published:** 2025-12-02

**Authors:** Panagiotis Tsiplakos, Andreas Panagopoulos, Konstantinos Katsanos, Nikolaos Parchas, Evangelia Christodoulou, John Lakoumentas, Dimitris Karnabatidis, John Gliatis

**Affiliations:** 1 Orthopaedics, School of Medicine, University of Patras, Patras, GRC; 2 Interventional Radiology, Patras University Hospital, Patras, GRC; 3 Orthopaedic Surgery, General Hospital of Leros, Leros, GRC; 4 Medical Physics, School of Medicine, University of Patras, Patras, GRC

**Keywords:** cooled radiofrequency ablation, cryoneurolysis, genicular nerves, knee osteoarthritis, persistent knee pain

## Abstract

Purpose: Knee osteoarthritis (KOA) is a common disease that significantly affects the quality of life of patients. Among several nonsurgical methods of symptomatic treatment, cooled radiofrequency ablation (CRFA) and cryoneurolysis (CRYO) have gained traction recently. This study compared the two methods in their capacity to reduce pain and improve clinical outcomes in patients with KOA.

Methods: This was a prospective, randomized, single-blinded clinical trial that included 25 patients with KOA in each group. The classic targets of the superior lateral and medial genicular and inferior medial genicular nerves were used, as well as the medial (retinacular) genicular branch from the vastus intermedius. Patients were evaluated using the Numerical Pain Scale (NRPS), the Knee Injury and Osteoarthritis Outcome Score (KOOS), and the Oxford Knee Score (OKS) at baseline and at one, three, and six months post-intervention.

Results: Both methods were able to reduce pain and display improved clinical outcomes in all three post-intervention evaluations. However, at the six-month mark, CRFA showed a clear advantage: NRPS decreased from a baseline mean of 7 to 3.1 (compared to 6.2 for cryoneurolysis); KOOS improved from a baseline mean of 45.4 to 64 (compared to 56 for cryoneurolysis); OKS improved from a baseline mean of 20 to 33 (compared to 26 for cryoneurolysis). No serious procedure-related adverse events were reported.

Conclusions: Both CRFA and cryoneurolysis of the genicular nerves are effective treatment options for KOA symptoms. However, CRFA displays a more durable therapeutic effect after the one-month period, with a clear advantage at the six-month evaluation.

## Introduction

Knee osteoarthritis (KOA) is a degenerative joint disease that affects the subchondral bone, synovium, menisci, periarticular muscles, ligaments, and tendons, resulting in cartilage loss, osteophyte formation, joint laxity, muscle wasting, synovial inflammation, and subchondral bone remodeling [1]. KOA has a high prevalence and is one of the leading causes of disability according to the 2015 Global Burden of Disease study [2]. Not only is KOA a common and debilitating disease, but it also displays a comorbidity rate of 68-85%, forcing an adaptation to conservative management strategies [3]. Notwithstanding that total knee arthroplasty (TKA) can provide a better quality of life for patients with end-stage KOA, it is important to note that a significant percentage of patients still report dissatisfaction [4]. Several factors are linked to a poor postoperative outcome, regardless of surgical technique, including mental health problems, lower back pain, overestimated preoperative expectations, and severe postoperative pain [5]. However, the slow progressive nature of knee osteoarthritis allows the utilization of drugs and minimally invasive methods for a considerable percentage of patients [6,7]. Under the umbrella of such treatment options, cooled radiofrequency ablation (CRFA) and cryoneurolysis (CRYO) have emerged.

Radiofrequency ablation (RFA) uses a probe that emits radiofrequency energy, thus ionizing and heating tissues in proximity. Chafing of these tissues prohibits the ionizing energy from spreading, creating a small radius of therapy. Cooled radiofrequency ablation (CRFA) adds a water supply chamber to the tip of the probe to provide a cooling effect. This prevents tissue chafing, allowing the radiofrequency energy to spread further. That way, although the probes operate at a temperature of 60°C, the surrounding tissues can reach a temperature of 80°C, and the radius of therapeutic heating is much larger. This method can be used to cause thermal nerve degradation and has implications in pain relief for patients suffering from KOA [8,9]. In most CRFA studies, the targeted nerves are the superior lateral genicular (SLGN), superior medial genicular (SMGN), and inferior medial genicular (IMGN), but in some studies, the medial retinacular genicular nerve from the vastus intermedius (MRGN) is also targeted [10,11].

Cryoneurolysis is the process of inducing Wallerian degeneration of the peripheral nerves by exposing them to low temperatures of -20 to -80°C. This is described as a second-degree injury to the nerve, leading to reversible damage of the axon, whereas other parts of the nerve remain intact. This allows potential regeneration and reinnervation of the target area. Studies regarding cryoneurolysis treatment for KOA thus far have targeted the infrapatellar branch of the saphenous nerve (IPBSN), a sensory nerve providing innervation of the anterior and inferior parts of the knee capsule, and the anterior femoral cutaneous nerve (AFCN) [12-16].

As the current literature suggests, both of these methods are effective in controlling KOA pain in the short term [11-18]. However, the targeting nerves are somewhat different, so a direct comparison of CRFA and CRYO efficacy cannot be confirmed. This study aims to provide a common targeting technique for these methods, so that a safer conclusion can be made.

The primary objective of the study was to evaluate the efficacy of CRFA and CRYO at one-, three-, and six-month post-intervention in patients with painful KOA using the Numerical Rating Pain Scale (NRPS).

The secondary objective was the comparison of safety and tolerability of the two interventions, as well as the clinical outcome at one-, three-, and six-month post-intervention, using the Knee Injury and Osteoarthritis Outcome Score (KOOS) and the Oxford Knee Score (OKS).

## Materials and methods

**Figure 1:**
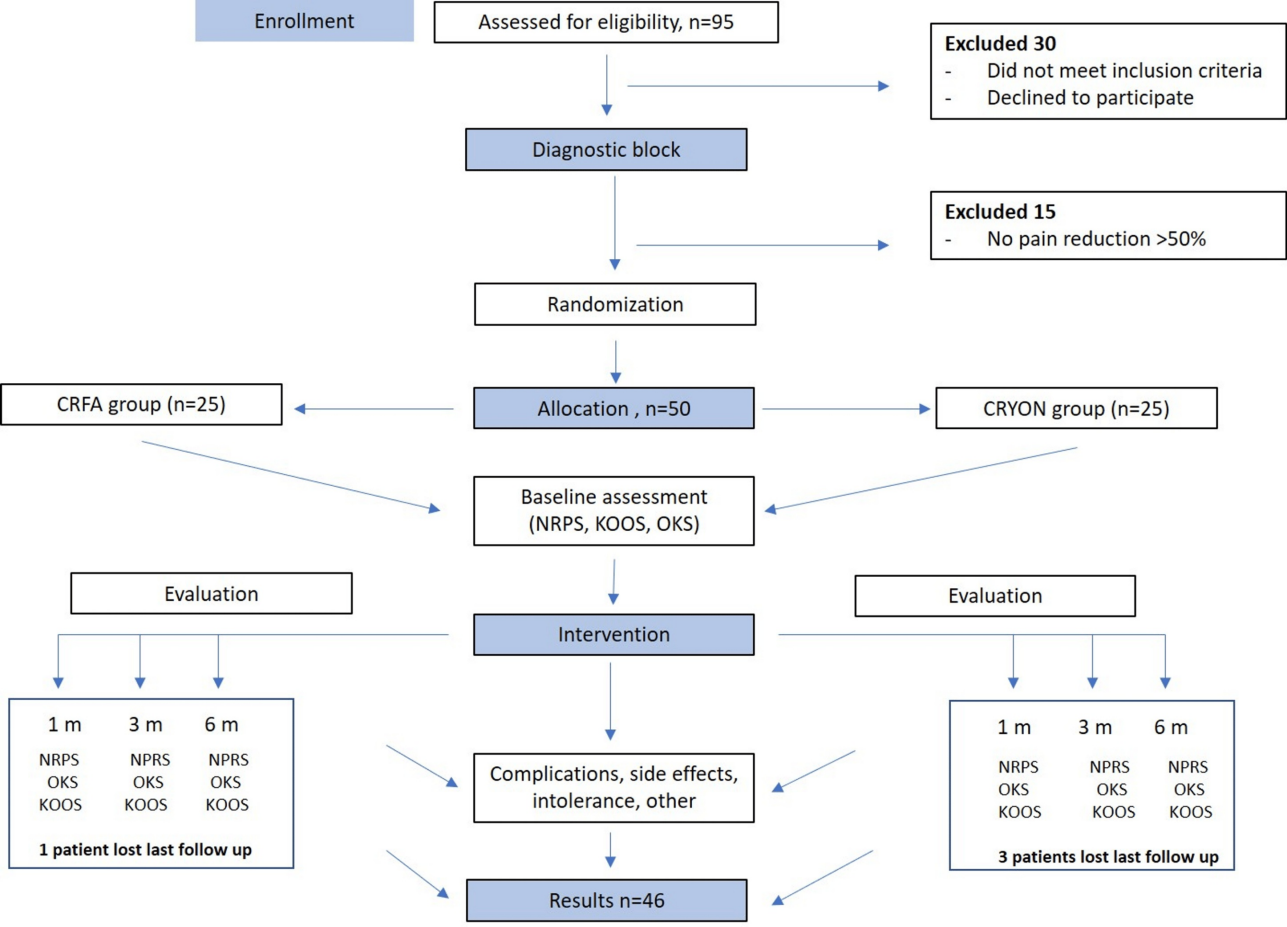
Flowchart of the study. CRFA: Cooled RadioFrequency Ablation, CRYON: Cryoneurolysis, NRPS: Numerical Pain Rating Scale, KOOS: Knee Osteoarthritis Outcome Score, OKS: Oxford Knee Score.

This study was a prospective, single-blinded RCT involving 25 patients in each group. The main hypothesis was that both methods would provide substantial pain relief compared to baseline values and that both methods would be effective and tolerable in the intermediate management of KOA (Figure 1).

The study was approved by the institutional review board (IRB) of University Hospital of Patras, Greece (11846/05/10/2021), and was conducted in accordance with all applicable laws and regulations as specified in the International Conference on Harmonization Guideline for Good Clinical Practice and the Declaration of Helsinki. Clinical trial registration was obtained (ISRCTN87455770 (https://doi.org/10.1186/ISRCTN87455770), registered March 29, 2022 (first patient recruited August 31, 2022)).

A total of 95 patients who met the NICE clinical criteria [19] of primary KOA for one or both knees were initially screened for eligibility: age >45; activity-related joint pain; no morning joint stiffness; radiological confirmation of grade ≥2 KOA according to the Kellgren and Lawrence classification [20]; minimum duration of symptoms of six months; and pain intensity ≥4 on the NRPS.

Seven patients declined to participate, and 23 were excluded before the diagnostic block for various reasons, including inflammatory arthritis; intra-articular injections in the last three months; significant structural deformities affecting knee function; BMI ≥40 kg/m²; serious illness; unstable psychiatric disease; coagulopathy or bleeding disorders; and active systemic or local infection.

Sixty-five patients were considered eligible for inclusion and underwent a diagnostic genicular block at the office using a portable ultrasound device, targeting the three classic genicular nerves using the genicular arteries as landmarks, whereas the medial (retinacular) genicular branch from the vastus intermedius (MRGN) was targeted approximately 5 cm above the superior patella pole. Patients were then asked to rate the percentage reduction in their knee pain while performing walking, deep squatting, and other maneuvers that typically provoked their pain during the next 30 minutes in the office. Fifteen patients experienced no change in perceived pain and were excluded from the study.

Fifty patients who fulfilled the inclusion criteria were informed about CRFA and CRYO, and after a detailed explanation of the study protocol, informed consent was obtained. A randomization sequence was created by an independent investigator (J.L.) using Stata 9.0 (StataCorp, College Station, Texas) statistical software and was stratified with a 1:1 allocation ratio using random block sizes of 2, 4, and 6; the data were stored on a computer and were available on the day of the intervention. For each intervention, this independent investigator handed an opaque envelope to the coordinating nurse just before the procedure. The patients, relatives, investigators, nurses, and all relevant personnel were blinded to the upcoming intervention. Both generators of CRFA and CRYO were available. The surgeon (AP), who performed all the procedures, was sterile and ready to apply the intervention. After opening the envelope, the upcoming procedure was unblinded for everyone.

Patients were placed on the radiolucent table at the Department of Interventional Radiology in a supine position with a bolster under the knee to produce 30° of flexion; this position flattens the suprapatellar joint space, thus minimizing the chance of intra-articular needle placement, and also allows for an unobstructed lateral view of the knee. The treated knee was draped and sterilized in a standard manner. Patients were continuously monitored and administered conscious sedation (1-2 mg IV midazolam and/or fentanyl 25-100 mcg IV) and supplemental oxygen by an experienced anesthesiologist. Fluoroscopic images were obtained to align the femur in an anteroposterior (AP) view to target the genicular nerves at locations above the femoral condyles, and for the IMGN, a greater caudal tilt of the fluoroscope was obtained to square off the tibial plateau for an appropriate AP view.

**Figure 2:**
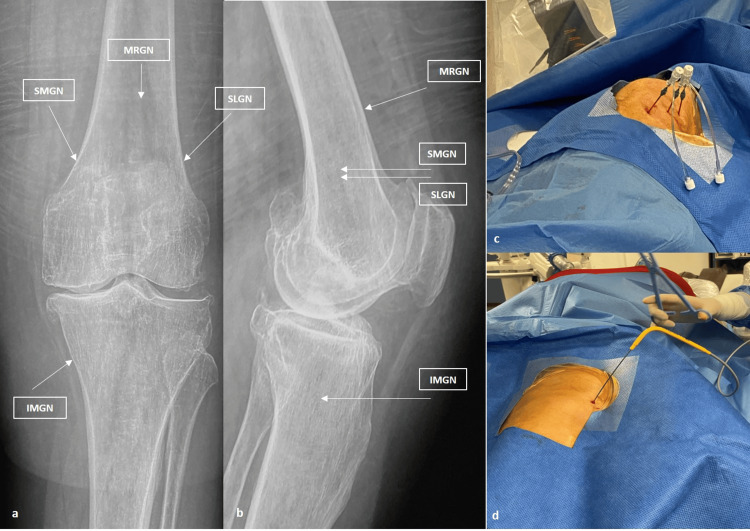
Surgical technique of ablation and targeting points. (a) Anteroposterior and (b) lateral X-rays of the knee indicating the target points of the genicular nerves; (c) intraoperative photo of the CRFA and (b) CRYO application in respect. SLGN: superior lateral genicular nerve, SMGN: superior medial genicular nerve, IMGN: inferior medial genicular nerve MRGN: medial (retinacular) genicular branch from vastus intermedius.

The nerve targets for the treatment were the classic radiological targets proposed by Choi et al. [21], McCormick et al. [22], and Conger et al. [23], involving the superior lateral genicular nerve (SLGN), superior medial genicular nerve (SMGN), and inferior medial genicular nerve (IMGN), as well as the medial (retinacular) genicular branch from the vastus intermedius (MRGN) for both cryoneurolysis and CRFA. SLGN and SMGN were located at the junction of the midpoint of the femoral shaft and the lateral or medial femoral condyle. IMGN was located at the junction of the midpoint of the tibial shaft and the medial tibial condyle. MRGN was located at the middle of the AP distance of the femur, 4-5 cm above the superior patella pole, as described by Wong et al. [11] (Figure 2).

For the CRFA technique, 2-3 mL of 1% lidocaine was used to anesthetize the skin and subcutaneous tissues before cannula insertion at each target site under fluoroscopic guidance. Thereafter, a 150 mm, 17-gauge introducer needle was placed to ablate the SLGN, SMGN, IMGN, and MRGN. One milliliter of 2% lidocaine was injected through the introducer needles to anesthetize the area before ablation. After placement of the introducer needle, an 18-gauge, internally cooled 4 mm active tip RFA electrode (Coolief, Halyard Health, Alpharetta, Georgia) was placed into the introducer needle, and the positioning was checked again using fluoroscopy. The introducer was connected to the COOLIEF* Cooled RF Advanced Generator that allows staggered start, stop, and adjustment of four independent channels. Motor nerve activity was excluded by testing at 2 Hz and 1 mA. The CRFA probes were advanced, and ablation was performed with lesion settings at 60 °C (80-90 °C adjacent tissue temperature) for 2.5 minutes.

For the CRYO technique, 2-3 mL of 1% lidocaine was used to anesthetize the skin and subcutaneous tissues before probe insertion at each target site under fluoroscopic guidance. A cryoneurolysis probe (ICESphere 1.5 CX, Galil Medical Ltd.) was inserted in the proximity of the four target points, guided by fluoroscopic visualization, as previously described. The VISUALL ICE™ generator (Boston Scientific, Marlborough) was used for cryoneurolysis and utilizes Argon as a coolant and Helium to thaw. The procedure was performed with a single freeze cycle: 30 seconds at an effect of 20% and 2 minutes and 30 seconds at 60% effect. After the freezing cycle, one minute of active thaw and one minute of passive thaw were used. The cryoneurolysis probe creates an ellipsoid lesion (ice-ball) that surrounds the active tip. The temperature gradient away from the probe increases significantly; the typical isotherm with the needle has a temperature of −40 °C in an area equivalent to 15 × 23 mm. At 23 × 29 mm, the temperature rises to −20 °C, and at 33 × 37 mm, the temperature is up to 0 °C. The thawing phase (two minutes) is not considered active treatment but is necessary for the ice-ball to melt gradually for later safe removal of the probe.

The active ablation time of cryoneurolysis (three minutes) is similar to that of CRFA (2.5 minutes) but with a different mechanism. The area of ablation is ellipsoid in both techniques, thus increasing the probability that a targeted sensory nerve will be captured in the “sphere” of tissues neuroablated. Finally, the “slow” effect of freezing (20% for 30 seconds and 2 minutes and 30 seconds at 60%) has already been proposed for genicular nerve ablation in patients with KOA [14] and is considered less destructive, as it aims at Wallerian nerve degeneration rather than nerve tissue destruction.

The patients then discontinued all other KOA treatments and pain medications, and they were evaluated at one, three, and six months using the NRPS, KOOS, and OKS by an orthopaedic resident (N.P.) who was not involved in the initial patient evaluation and was blinded to the applied treatment. The part of the KOOS questionnaire that involved sporting and recreational activities was not utilized, as the patient population was older and did not take part in high-level activities. The mean KOOS score was calculated using the other four sections of the score that were more relevant for the studied population. One patient from the CRFA group and two from the CRYO group died for reasons unrelated to the treatment. Another patient from the CRYO group lost two of the three re-examinations. A total of 46 patients (24 CRFA, 22 CRYO) were available for the final analysis.

Statistical analysis

For determining if there is a statistical difference in the scores between groups, first, repeated measures ANOVA (rmANOVA) was used. Then, a bivariate ANCOVA was run comparing the baseline results for each method against the results for each subsequent re-examination. The ANCOVA analysis, which combines ANOVA with linear regression, normalizes the subsequent measurements to the baseline and thus requires a much lower sample size compared to ANOVA, provided that the correlation estimates between the a priori and a posteriori scores are approximately 80-90%. Thus, we can achieve an 80% confidence level with a 5% significance level with a sample size of 24-45 [24].

For the rmANOVA analysis, all assumptions for all three tests were met, and, more specifically, Box’s test sig. (>0.05), Wilks-L sig. (<0.05), Mauchly’s sphericity test (<0.05), whereas all of Levene’s tests showed sig. (>0.05), with the exception of the KOOS sphericity test (0.13). However, the Epsilon Greenhouse-Geisser estimate was above 0.79 (0.822), and the Greenhouse-Geisser test sig. was 0.0. Furthermore, all ANCOVA assumptions were met for KOOS, OKS, and NRPS scores. Statistical difference of covariates of the pre-test measurements was checked with ANOVA (sig. >0.05), and data homogeneity was ensured with regression with group*time sig. also >0.05.

## Results

Forty-six patients were available for comprehensive analysis. There were no statistical differences in mean age, sex, duration of symptoms, BMI, and the mean value of pre-intervention NRPS. Most patients in both groups had Kellgren-Lawrence KOA stage 2 or 3 (75% CRFA, 90% CRYO) (Table 1). Major complications, such as neurovascular injuries or infections, were not reported. Most patients had minor swelling around the knee joint for the first days after the treatment, which was easily managed with elastic bandage compression and application of ice.

**Table 1:** Demographic data of the included patients The mean duration of symptoms using the chi-square test had an effect size (Cohen’s d) of 0.012, and 43 degrees of freedom (Welch’s df) CRFA: cooled radiofrequency ablation, CRYO: cryoneurolysis, SD: standard deviation, BMI: body mass index, KOA: knee osteoarthritis, NRPS: numerical rating pain scale.

Variables	CRFA	CRYO	P-value
Total patient count	24	22	
Mean patient age (SD), years	67.17 (10.82)	69.05 (8.7)	0.25 (t-test)
Female	17	14	
Male	7	8	
Mean body mass index, BMI (SD)	29.1 (3.55)	29.78 (4.6)	0.45 (t-test)
Mean duration of symptoms (SD)	59.17 (6.34)	59.09 (6.63)	0.3 (chi-sq)
Medication for knee pain	20	19	
KOA (Kelgren Lawrence classification)			
Grade 1	0	0	
Grade 2	7	9	
Grade 3	11	11	
Grade 4	4	2	
Mean NRPS score prior to treatment (SD)	6.83 (1.37)	7.32 (1.2)	0.55 (t-test)

**Figure 3:**
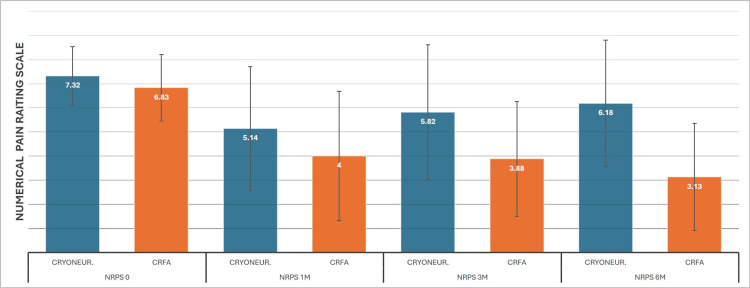
Cooled RadioFrequency Ablation (CRFA) versus Cryoneurolysis (CRYONEUR) measurements on Numerical Pain Rating Scale (NPRS)

**Figure 4:**
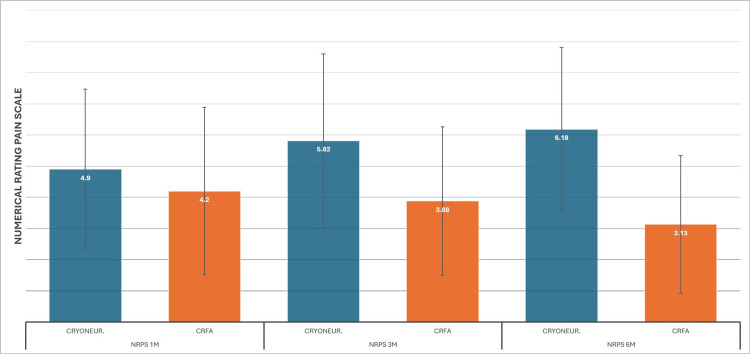
Cooled RadioFrequency Ablation (CRFA) versus Cryoneurolysis (CRYONEUR) ANCOVA results for Numerical Pain Rating Scale (NPRS)

The primary objective of the study was to evaluate the efficacy of CRFA and CRYO at one, three, and six months post-intervention using the NRPS. rmANOVA analysis for the CRYO patients started with a 7.3 (SD±1.21) NRPS baseline, with 5.1 (SD±2.56), 5.8 (SD±2.78), and 6.1 (SD±2.63) for the subsequent measurements. CRYO had its biggest effect at one month and offered a statistically significant improvement, although the effect was considerably reduced at six months. On the other hand, CRFA scores were 6.8 (SD±1.37), 4 (SD±2.68), 3.8 (SD±2.38), and 3.1 (SD±2.21), respectively, showing a consistently significant improvement in knee pain (Figure 3). The ANCOVA analysis equalized the baseline mean to 7. On this test, however, a significantly higher reduction in pain was observed for CRFA from the first month (4.2 to 4.9), which was even higher in subsequent examinations: 3.9 to 5.8 at three months and 3.1 to 6.2 at six months (Figure 4).

KOOS evaluation

**Figure 5:**
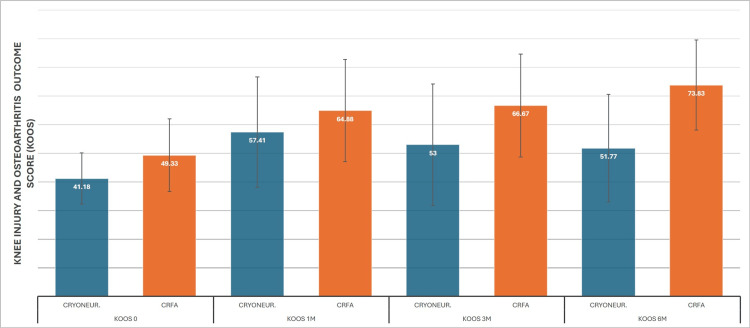
Cooled RadioFrequency Ablation (CRFA) versus Cryoneurolysis (CRYONEUR) mean Knee Injury and Osteoarthritis Outcome Score (KOOS) measurements

**Figure 6:**
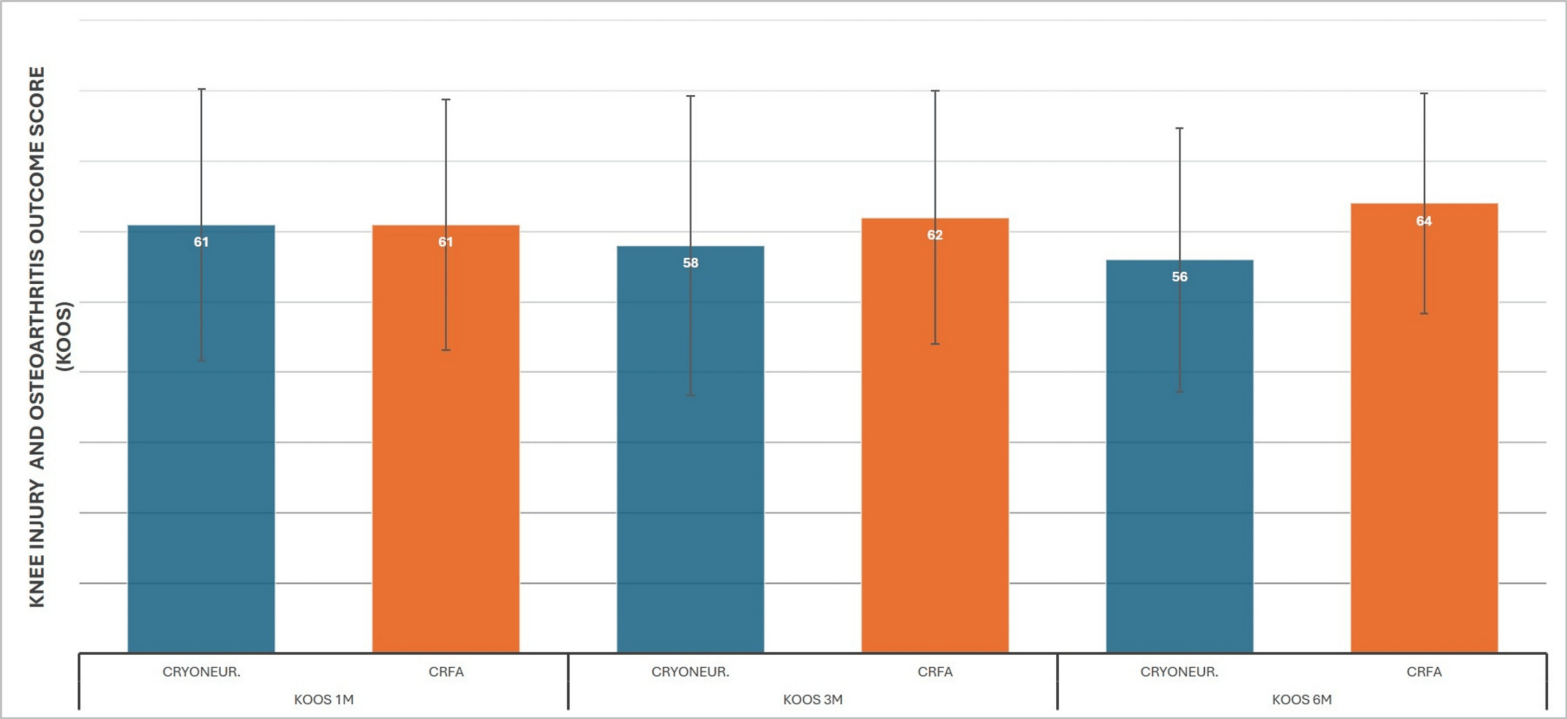
Cooled RadioFrequency Ablation (CRFA) versus Cryoneurolysis (CRYONEUR) mean Knee Injury and Osteoarthritis Outcome Score (KOOS) ANCOVA measurements

Means for CRYO pre-treatment and at one-, three-, and six-month re-examinations were 41 (SD±8.9), 57 (SD±19.3), 53 (SD±21.23), and 51 (SD±18.7), respectively. Statistically significant improvement was observed from baseline at all three re-examinations; however, there was a significant decline between the first and third month, as well as a decline from three to six months, although this was not statistically significant. On the other hand, CRFA results were 49 (SD±12.7), 64 (SD±17.8), 66 (SD±17.9), and 73 (SD±15.6) from time 0 to six months, showing an improvement trend across all dates (Figure 5). ANCOVA reveals more clearly the advantage of CRFA over CRYO, with the baseline mean normalized to 45.4. At the first-month mark, means are equal at 61 for each test; however, at the three- and six-month marks, CRFA demonstrates a clear therapeutic advantage with 62 vs 58 and 64 vs 56 normalized means (Figure 6).

OKS evaluation

**Figure 7:**
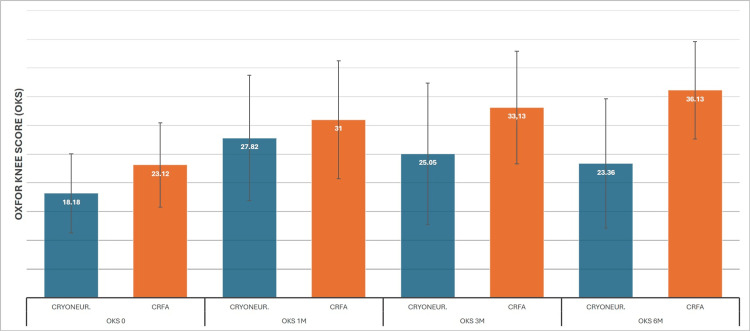
Cooled RadioFrequency Ablation (CRFA) versus Cryoneurolysis (CRYONEUR) mean Oxford Knee Score (OKS) measurements

**Figure 8:**
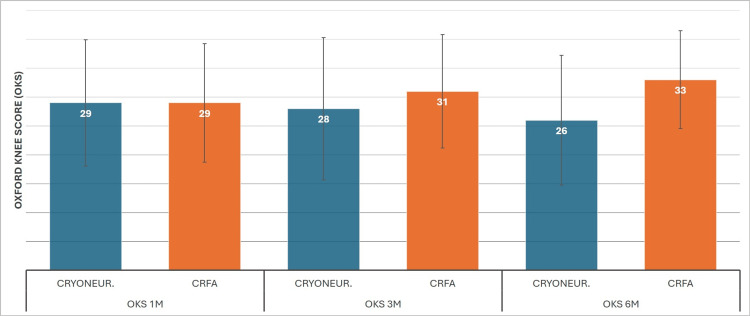
Cooled RadioFrequency Ablation (CRFA) versus Cryoneurolysis (CRYONEUR) mean Oxford Knee Score (OKS) ANCOVA measurements

Starting from time 0, the data for CRYO were 18 (SD±6.8), 27 (SD±10.9), 25 (SD±12.3), and 23 (SD±11.2). We can observe the same trend as with the KOOS measurement, with the initial highest mean score for the patient undergoing cryoneurolysis treatment at one month and a gradual regression on later dates, although with a significant six-month improvement compared to the baseline. The same pattern with the KOOS score is also apparent for the CRFA, with means of 23 (SD±7.3), 31 (SD±10.2), 33 (SD±9.8), and 36 (SD±8.48) (Figure 7). ANCOVA again showed superior results for CRFA after the first month post-treatment. Baseline mean was calculated as 20, and the first month normalization was to 29 for both treatments. At three months, CRFA had 31 versus 28, and at six months, 33 versus 26 (Figure 8).

All patients were contacted (by phone) at one and two years after the intervention, and they were asked if their symptoms had deteriorated and if they had finally undergone a total knee replacement. Conversion to total knee arthroplasty was necessary in 6/22 (27%) patients in the CRYO group and in 7/24 (29%) in the CRFA group after a mean period of 17.5 months after the procedure. These included all six patients with type IV arthritis and seven with type III arthritis.

## Discussion

The main takeaway from this clinical trial is that CRFA provides a more durable improvement compared to CRYO for the treatment of KOA symptoms. This was expected, as CRFA thermally ablates the nerves, whereas cryoneurolysis leads to Wallerian degeneration as stated above, allowing the nerves to regenerate. Moreover, both methods provided a statistically significant improvement in KOA symptoms, which is consistent with currently available clinical trials.

In 2017, Radnovich et al [13] conducted a clinical trial with 180 patients (n=121 cryoneurolysis and n=59 sham treatment). They used visualization and palpation to create a treatment line along the infrapatellar branch of the saphenous nerve (IPBSN). Patients who received cryoneurolysis had a statistically significant pain reduction using the pain subscale of the WOMAC score up to 90 days post-treatment. Patients deemed WOMAC pain responders at day 120 continued to have a statistically significant improvement even at day 150, but not at day 180. These results imply that the treatment effect of cryoneurolysis may degrade over time, something that the authors also mentioned. Nygaard NB et al [14] recently reported a cryoneurolysis versus sham double-blinded randomized controlled trial in 87 patients targeting the anterior femoral cutaneous nerve (AFCN) and the IPBSN. They found no difference in the primary outcome, which was the difference in average pain at 14 days post-intervention.

Randomized trials suggest that CRFA of the genicular nerves not only provides favorable outcomes at six months post-intervention [17, 25-27], but could also provide relief for up to 12 months [18, 28]. In an observational extension of an RCT evaluating the treatment effect of CRFA during a 12-month period, Lyman et al [29] demonstrated that patients who respond to CRFA treatment can have a durable outcome extending up to 24 months. Similar results were observed by Hunter et al [30], where patients demonstrated pain relief that lasted 24 months. These results imply that CRFA could theoretically provide a more durable treatment outcome than CRYO for KOA. However, no direct comparison of the methods could be established, as most CRFA studies targeted the common genicular nerves, whereas cryoneurolysis studies targeted the IPBSN and the AFCN.

To make the comparison more reliable in our study, we targeted the same genicular nerves in both CRFA and CRYO interventions. Post-intervention evaluations were also performed using the same clinical scores and questionnaires. In our study, both methods provided statistically significant improvement in pain and clinical outcomes for up to six months. In the first month post-intervention, cryoneurolysis demonstrated a significant pain reduction using rmANOVA analysis (NRPS reduction from 7.3 to 5.1) and improvement in clinical outcomes (KOOS improvement from 41 to 57 and OKS from 18 to 27). However, at the third month post-intervention, the pain started to increase gradually (NRPS from 5.1 to 5.8), and the clinical outcomes started to deteriorate (KOOS from 57 to 53 and OKS from 27 to 25). The same trend was observed at the six-month evaluation. NRPS increased from 5.8 to 6.1, while KOOS and OKS decreased from 53 to 51 and from 25 to 23, respectively. Even though there is an improvement from baseline using cryoneurolysis (mean NRPS reduction of 1.2, mean KOOS improvement of 10, and mean OKS improvement of 5), the effects seemed to fade over time, implying a less durable outcome. Despite that, cryoneurolysis of the genicular nerves appeared to be an effective short-term treatment option for KOA in our clinical trial.

CRFA of the genicular nerves demonstrated a statistically significant improvement in pain and clinical outcomes for up to six months, and the results continued to improve rather than fade. More specifically, at the six-month evaluation, NRPS was reduced from a baseline of 6.8 to 3.1, KOOS improved from 49 to 73, and OKS showed similar improvement from 23 to 36. When the results for both techniques were normalized using ANCOVA, the difference in outcome durability remained evident at the six-month mark: NRPScryo=6.2 versus NRPScrfa=3.1, KOOScryo=56 versus KOOScrfa=64, and OKScryo=26 versus OKScrfa=33.

Strengths and limitations

This is a single-blinded randomized controlled trial with a follow-up period of six months. Our main strength is that the targeting techniques and methods used were homogenized in the two groups to allow safer conclusions. However, we have to acknowledge two important limitations. The effectiveness of these treatments relies heavily on the accurate targeting of the aforementioned nerves. Inaccurate targeting in some patients is possible even when using the same detailed technique, introducing an element of variability in the results. The number of patients is another limitation, as it provides us with an 80% confidence level. More research is clearly needed to allow for more robust conclusions.

## Conclusions

This was a single-blinded randomized controlled trial with three follow-up examinations per patient over a period of six months. The sample size allowed a statistical power of 80%. The targeting of the nerves was conducted in accordance with old, proven clinical reports. The method of applying both CRYO and CRFA was the same according to the manufacturer's protocols. However, technical limitations exist, as the effectiveness of these treatments relies heavily on accurate targeting of the genicular nerves and also on the fact that we are not able to ablate all the nerves of the knee joint capsule. Our study showed that both CRFA and CRYO of the genicular nerves are effective treatment options for the symptomatic management of KOA symptoms. However, CRFA displays a more durable therapeutic effect after the one-month period, with a clear advantage at the sixth-month evaluation.
